# Retraction: Thoracic Vertebral Actinomycosis Secondary to a Pulmonary Origin

**DOI:** 10.7759/cureus.r10

**Published:** 2018-01-22

**Authors:** Nqobile S Thango, A Kamat, Mohamed Ben Husein, David Welsh

**Affiliations:** 1 Department of Neurosurgery, Groote Schuur Hospital, University of Cape Town; 2 Medical Officer, Groote Schuur Hospital

This article has been retracted at the request of the University of Cape Town and the Cureus Editor-in-Chief. Cureus editorial staff detected several irregularities with this article and contacted the submitting author and his corresponding department heads. While the submitting author did not respond to this initial query, the University of Cape Town initiated a full investigation. After a comprehensive investigation by a Special Investigation Committee at UCT, it was determined that all authors associated with this article are guilty of both plagiarism and falsification of images.

Figure 3 in the article purports to depict human brain histology to complement the content of the article but, in fact, is a picture of mouse brain histology (6-week-old male Swiss Webster mouse (Mus musculus)) taken from an open access database. The image is clearly plagiarised. It also is a false picture that does not represent the content of the article.

The hyperlink to the relevant image is "https://www.askjpc.org/wsco/wsc_showcase2.php?id=523"

The authors responded to the preliminary report variously denying culpability, knowledge of improper use of images or failure to acknowledge sources. However, these denials are not relevant to whether plagiarism exists in the article. The allegation of fabrication or falsification can also be established without difficulty. The article states that the image came from GSH databases and represents human brain histology of the patient concerned – it does not. 

 

A key condition of article submission is that authors must explicitly declare that their work is original and has not appeared in a publication elsewhere. Re-use of any data or images must be appropriately cited. As such this article represents a severe abuse of the scientific publishing system. The Cureus Journal of Medical Science takes a very strong view on this matter and we apologize that this was not detected during the submission process. The institutions of all authors have been notified and all authors are hereby banned from submitting further work to The Cureus Journal of Medical Science.

